# Newly Discovered Mechanisms of Antibiotic Self-Resistance with Multiple Enzymes Acting at Different Locations and Stages

**DOI:** 10.3390/antibiotics12010035

**Published:** 2022-12-26

**Authors:** Xiaorong Chen, Hai-Xue Pan, Gong-Li Tang

**Affiliations:** 1 School of Chemistry and Materials Science, Hangzhou Institute for Advanced Study, University of Chinese Academy of Sciences, 1 Sub-Lane Xiangshan, Hangzhou 310024, China; 2 State Key Laboratory of Bioorganic and Natural Products Chemistry, Shanghai Institute of Organic Chemistry, University of Chinese Academy of Sciences, Chinese Academy of Sciences, Shanghai 200032, China

**Keywords:** self-resistance, natural products, biosynthetic gene clusters, temporal-spatial shielding, intracellular multi-level resistance

## Abstract

Self-resistance determinants are essential for the biosynthesis of bioactive natural products and are closely related to drug resistance in clinical settings. The study of self-resistance mechanisms has long moved forward on the discovery of new resistance genes and the characterization of enzymatic reactions catalyzed by these proteins. However, as more examples of self-resistance have been reported, it has been revealed that the enzymatic reactions contribute to self-protection are not confined to the cellular location where the final toxic compounds are present. In this review, we summarize representative examples of self-resistance mechanisms for bioactive natural products functional at different cell locations to explore the models of resistance strategies involved. Moreover, we also highlight those resistance determinants that are widespread in nature and describe the applications of self-resistance genes in natural product mining to interrogate the landscape of self-resistance genes in drug resistance-related new drug discovery.

## 1. Introduction

Natural products are small chemical compounds derived from secondary metabolites of animals, plants, and microorganisms with an array of biological activities. Since the discovery and clinical use of penicillin and streptomycin [1,2], natural products have been an important source of drugs and play a critical role in modern medicine and agricultural industries [2]. However, widespread use and misuse of these small chemical compounds has led to the rising emergence of antibiotic resistance, resulting in the gradual loss of efficacy of antibiotics in clinic and natural environment. Antibiotic resistance has become one of the greatest public health threats that humans will have to face in the coming decades [3,4]. According to a report from the UK government, the death toll caused by antibiotic resistance is estimated to be up to 10 million per year by 2050, with a cost of drug resistance to $100 trillion [5]. Therefore, there is an urgent need for uncovering the resistance mechanisms in pathogens and developing new compounds with novel modes of action [6,7,8].

Generally, the clinical antibiotic resistance in human pathogens belongs to acquired resistance, with only a small fraction under the category of innate resistance. The origins of these acquired resistance genes have been traced to antibiotic-producers in natural environments [9,10]. In antibiotic-producing microbes, self-resistance is a prerequisite for the synthesis of antibiotics [11]. Antibiotic biosynthetic gene clusters (BGCs) contain one or more resistance genes to achieve self-protection, and these genes are considered to be the reservoirs of resistance genes, which may transfer to human pathogens by conjugation, transformation or transduction [9,10,12,13]. Consequently, the elucidation of self-resistance mechanisms from antibiotic-producing microbes will not only reveal the action model of antibiotics and guide the discovery of new natural products, but also provide key clues for the studies of clinical antibiotic resistance [8,10,13,14].

To avoid suicide, antibiotic-producers have developed several mechanisms, including efflux pumps, chemical modification, prodrugs, compound sequestration, (sub)cellular location, target modification, and damage repair to shield the toxicity of antibiotics, thereby achieving self-protection [6]. Different strategies are adapted depending on the structure of antibiotics, molecular target and producer species [8]. For instance, within the enediyne producers, apoproteins are known to afford self-protection to the producers by binding the nine-membered enediyne chromophore, whereas the strategies of chemical modification guided by self-sacrifice proteins and sequestration mediated by drug binding proteins are utilized for the detoxification of ten-membered enediynes [15,16]. To prevent self-harm from bleomycin, its producer *Steptomyces verticillus* employs bleomycin N-acetylation and sequestration mechanisms to protect itself. The antitumor agent mitomycin C producer, *Steptomyces lavendulae*, has developed several mechanisms, including prodrug, efflux pump, drug sequestration, and reoxidation of the active reduced mitomycin C to ensure self-resistance [15].

With sustained advances in the study of self-resistance mechanisms, researchers have found that the assembly of antibiotics and self-resistance do not always occur at the same cellular location. In some cases, self-resistance is achieved through enzymatic reaction occurring at different timing and location [17,18,19]. Although the self-protection mechanisms in antibiotic-producing microbes and application of self-resistance genes in natural product mining have been described in previous reviews [6], the topic of self-resistance in microbes that synthesized final active drugs at different cell locations has never before been reviewed in its entirety. In addition, self-resistance genes that widely prevalent in nature were not described in previous reviews. Therefore, this contribution will provide an overview of newly-discovered self-resistance enzymes for natural products functional at different cell locations, explore the models of resistance strategies involved, describe those resistance determinants that are widespread in nature and the applications in natural product mining, and interrogate the landscape of self-resistance genes in combating drug resistance and future applications in new drug discovery. The self-resistance genes were selected as the keywords for the search in Google Scholar from 2016 to 2022.

**Figure 1:**
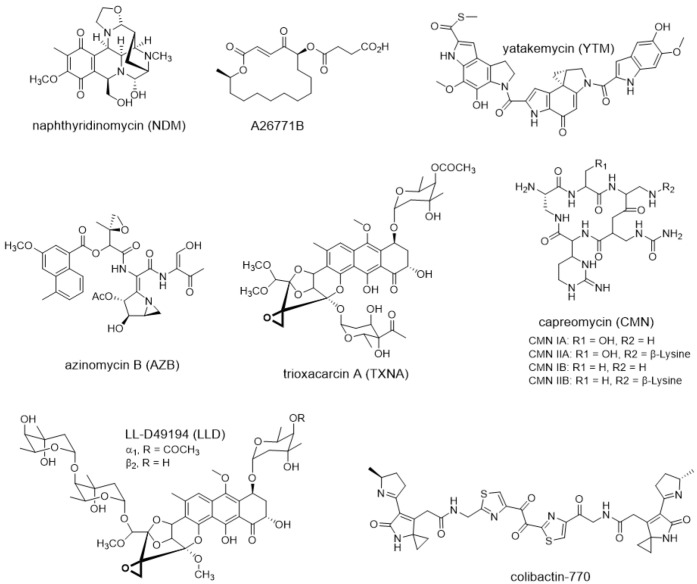
Representative natural products that employ temporal-spatial shielding or intracellular multi-level resistance model during biosynthesis.

In this review, we focus on new representative examples (Figure 1) of self-resistance mechanisms of bioactive natural products, aiming to provide a perspective on the ties between toxic antibiotics and self-resistance from timing and location. We present two models of self-resistance strategies: temporal-spatial shielding and non-spatial shielding–intracellular multi-level resistance. As for the intracellular multi-level resistance model, both of the enzymatic reactions catalyzed by resistance proteins and the assembly of toxic natural products occur inside cells. Instead, in the temporal-spatial shielding model, the final toxic compounds are produced extracellularly by secreted proteins, and self-resistance is achieved through enzymatic reactions occurring at different timing and location. Moreover, we also highlight those resistance determinants that are widely spread in bacteria and describe examples of the use of self-resistance genes to guide the discovery of new natural products. Taken together, we expect to provide some new insight on the role of resistance genes in natural product biosynthesis and in response to clinical resistance.

## 2. Temporal-Spatial Shielding Resistance

A drug molecule does not function unless it binds to its target. Microbes that synthesize antibiotics have developed a defensive strategy characterized as temporal-spatial shielding in order to prevent their own cytotoxic molecules binding to the target within the cells. The temporal-spatial shielding is a derivative of a common prodrug strategy, and the most significant difference between them is the presence or absence of secreted protein responsible for inert drug activation. In temporal-spatial shielding mode, antibiotics are synthesized as inert drugs and then transported to the extracellular space, followed by activation with enzymatic reactions catalyzed by secreted proteins, which contributes to the spatial shielding of drug activity. Moreover, cytosol-located resistance proteins will immediately inactivate the pharmacophore of synthetic intermediate or detoxify the final active drug that entered into the cell via passive diffusion, thus protecting the antibiotic producing microorganisms from timing. In fact, similar space shielding patterns are occasionally adopted to biosynthesize some aminoglycoside, macrolide and nonribosomal peptide antibiotics [11,20,21]. In those cases, the activation of chemically modified prodrugs is catalyzed by membrane-bound enzymes via the hydrolysis of the modified groups such as phosphate groups and leader peptides. However, in the temporal-spatial shielding model, the inert prodrugs are transported across the membrane and further activated by the secreted proteins in extracellular space.

**Figure 2:**
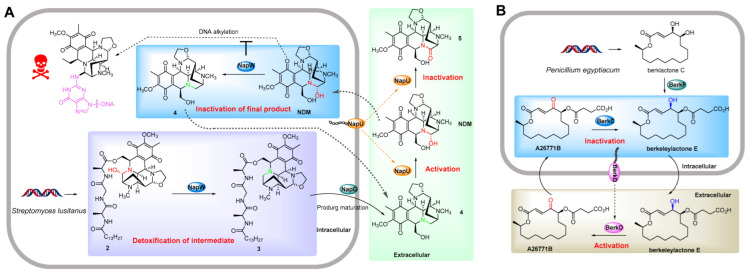
Examples of temporal-spatial shielding resistance model. (**A**) Self-resistance mechanisms of NDM producer Streptomyces lusitanus. Cytosol-located short-chain dehydrogenase/reductase NapW confers temporal shielding of NDM cytotoxicity by detoxification of biosynthetic intermediate and inactivation of re-entered final product. The membrane protease NapG couples with the secreted oxidoreductase NapU contributes to spatial shielding of NDM activity by prodrug maturation, NDM activation and inactivation. (**B**) Self-resistance mechanisms of A26771B producer Penicillium turbatum. Activation of the final product A26771B in extracellular space by the secreted oxidoreductase BerkD ensures the safety of producer via spatial shielding. Intracellular A26771B inactivation by cytoplasmic short-chain reductase BerkC and the subsequent recycling of the products control the self-harm of its producer from time.

A well-studied example of temporal-spatial shielding mode is the NapW/NapU self-protection system in naphthyridinomycin (NDM, **1**) biosynthesis, in which NapU is responsible for extracellular activation and NapW contributes to intracellular detoxification [17,18]. NDM, a member of tetrahydroisoquinoline alkaloids with complex hexacyclic structure, exhibits excellent antitumor and antimicrobial activities [22,23]. Its prominent biological activity is derived from the formation of electrophilic iminium species by eliminating C-7 hydroxyl, which covalently alkylates the N2 residue of guanine residing in the minor groove, forming DNA lesions that threaten cellular livelihood (Figure 2A). In the NDM biosynthetic pathway, cytosol-located short-chain dehydrogenase/reductase NapW is responsible for reducing the hemiaminal pharmacophore of intermediate **2** to generate the non-toxic **3** (Figure 2A), which ensures the harmlessness of following products and facilitates the biosynthetic process [18]. The resulting intermediate **3** is treated with a membrane protease NapG to produce the matured prodrug **4**, which is then transported out of the cell and activated by the secreted oxidoreductase NapU to generate the final product NDM (Figure 2A). Moreover, NapU catalyzes the overoxidative inactivation of NDM into **5** to control the extracellular concentration of antibiotic and reduce damage to the producer cell [17]. Beyond that, the re-entered final active drug NDM is also inactivated with the reductive reaction of NapW (Figure 2A) [18]. The mechanism of NDM inactivation is completely different between intracellular and extracellular. The intracellular detoxification is achieved by reduction reaction and the extracellular by oxidation [17,18], suggesting that new enzyme reactions are involved in pharmacophore modification beyond hydrolysis.

Compared to prokaryotes, the compartmentalized biosynthesis of natural products is more frequently used by eukaryotes to isolate the toxicity of substances, because the latter have discrete organelles to partition biosynthetic components orderly and efficiently [24]. An excellent example is the compartmentalized biosynthesis of fungal mycophenolic acid, which sequesters the inhibition of mycophenolic acid on inosine-5-monophosphate dehydrogenase away from the nucleus of fungi, thereby ensuring the normal synthesis of DNA [24,25]. However, there are few reports on self-resistance mediated by enzyme-catalyzed temporal-spatial shielding in the biosynthesis of eukaryotic natural products. Recent studies of self-protection strategies against fungal macrolides revealed that this self-resistance mode is involved in the biosynthesis of A26771B [19]. A26771B is a 16-membered fungal macrolide antibiotic isolated from *Penicillium turbatum*, and contains a succinate moiety and an unusual γ-keto-α,β-unsaturated carboxyl, among which the γ-keto group is the pharmacophore (Figure 2B) [26,27,28]. It exhibits prominent biological activity against Gram-positive bacteria and fungi, although with an unknown mode of action [26]. To biosynthesize A26771B efficiently, its producer utilizes a strategy of intracellular reduction and extracellular oxidation for reversible conversion between ketone and alcohol, thus protecting itself from the cytotoxic effect of A26771B (Figure 2B) [19]. Intracellular acyltransferase BerkF catalyzes monosuccinylation of biosynthetic intermediate Berklactone C to generate the mature non-toxic prodrug Berkeleylactone E, which is then exported out of the cell and oxidized by secreted oxidoreductase BerkD to form the target antibiotic A26771B. Moreover, cytoplasmic short-chain reductase BerkC will reduce the γ-keto group of antibiotic A26771B immediately once it enters into the host cell (Figure 2B). Therefore, the producer employs enzymatic reactions occurring at different timing and location to avoid self-destruction, which is appeared for the first time in fungal antibiotic biosynthesis. Given that the γ-keto-α,β-unsaturated carboxyl serves as the pharmacophore for many fungal macrolide antibiotics, this reversible conversion between ketone and alcohol might be a generic self-resistance strategy in fungal macrolides biosynthesis. However, no more examples are currently available and therefore further studies will be necessary to verify this hypothesis.

The self-resistance strategy involving temporal-spatial shielding is widely recognized to apply a secreted protein that located in the extracellular space to catalyze the maturation of prodrugs, thereby sequestering the toxicity of the final products away from their intracellular targets. This spatial compartmentalization of biosynthetic steps is similar to the compartmentalized biosynthesis of fungal natural products, for which the cell membrane acts as the shield in isolation of toxic substances [24]. Fungal cells comprise an additional endo-membrane system, whereas prokaryotes only contain an exo-membrane system. Therefore, in order to minimize the potential toxicity of natural products and control the efficiency of biosynthetic machinery, prokaryotes employ secreted proteins to complete the biosynthesis of final toxic products outside the cell. In addition, this temporal-spatial shielding mode of self-protection represents an evolutionary advantage and has been occasionally adopted by fungi to reduce the consumption of endogenous resources [19].

## 3. Intracellular Multi-Level Resistance

**Figure 3:**
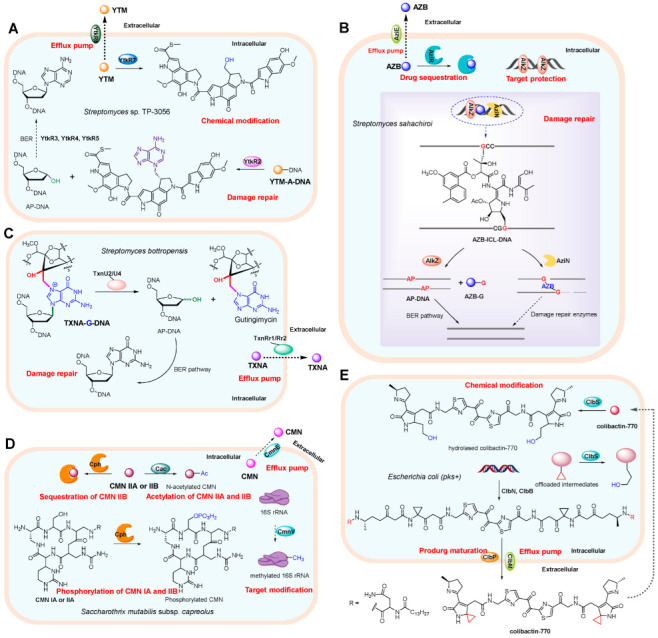
Examples of intracellular multi-level resistance model. (**A**) Self-resistance strategies employed by yatakemycin (YTM) producing strain *Streptomyces* sp. TP-3056. The producer is known to be protected by seven resistance genes that function in efflux pumps, chemical modification and damage repair. (**B**) Self-protection mechanisms of azinomycin B (AZB) producing strain Streptomyces sahachiroi. The drug-bind protein AziR, transmembrane export protein AziE, DNA damage repair enzymes AlkZ and AziN confer resistance to AZB in *Streptomyces sahachiroi* via drug sequestration, efflux pumps, target protection and damage repair, respectively. (**C**) Self-resistance strategies employed by trioxacarcin A (TXNA) producer *Streptomyces bottropensis*. To withstand the genotoxicity of TXN, the producer has evolved drug efflux pump and DNA damage repair mechanisms. (**D**) Multi-level resistance mechanism in capreomycin (CMN)-producing strain *Saccharothrix mutabilis* subsp. *capreolus*. N-acetyltransferase Cac is responsible for inactivating CMN IIA and IIB via acetylation, methyltransferase CmnU confers resistance to CMN by methylating 16S rRNA, phosphotransferase Cph plays role in the phosphorylation of CMN IA and IIA, and it also serves as an antibiotic sequester to inactivate CMN IIB by physical sequestration. (**E**) Multi-level resistance mechanism in colibactin-770-producing strain *Escherichia coli* (*pks+*). Nonribosomal peptide synthetases ClbN and ClbB prevent the formation of the active imine moiety during early stage of colibactin biosynthesis, inner-membrane transporter ClbM and peptidase ClbP are responsible for prodrug maturation and efflux. Moreover, the intracellular cyclopropane hydrolase ClbS abrogates cytotoxicity of the offloaded intermediates or re-entered colibactin.

Aside from synthesized in the form of prodrugs, which are subsequently generated to active compounds during or after excretion, natural products are often directly synthesized in active forms inside the cell. Therefore, some producers would generate a multi-level resistance strategy composed of several resistance mechanisms to protect themselves from the toxicity of intracellular antibiotics. Here, we enumerate some recently reported examples, including bacteria that produce yatakemycin (YTM), azinomycin B (AZB), trioxacarcin A (TXNA)/LL-D49194 (LLD), capreomycin (CMN), and colibactins (Figure 3).

YTM is a potent genotoxic agent belonging to the spirocyclopropylcyclohexadienone (SCPCHD) family natural products with remarkable cytotoxicity against various tumor cells and pathogenic fungi due to its DNA alkylating activity towards N3 position of adenine nucleobase and non-covalent CH-π interaction with deoxyribose group in DNA duplex [29,30,31,32,33].To counter its toxicity, the YTM-producer *Streptomyces* sp. TP-3056 has developed a multi-level resistance strategy for self-protection, including efflux and chemical modification of YTM and repair of DNA (Figure 3A). YtkR6 is homologous to the drug-resistance transporter ChaT1 [34], and thereby considered to perform the efflux of YTM to extracellular space, and serve as the primary detoxification mechanism. In addition, GyrI-like protein YtkR7 will eliminate the DNA alkylating activity through hydrolysis of cyclopropane warhead to minimize the YTM concentration inside cell [35]. Despite this, the active alkylating agent YTM can still covalently bind to the AT rich of DNA, thereby interrupting normal cellular process. DNA glycosylase YtkR2 is responsible for the remove of the 3-yatakemycinyladenine nucleobases through hydrolyzing the glycosidic bonds of YTM-DNA adducts, initiating the base excision repair pathway to complete the damage repair [36,37]. Structural analysis has revealed that, similar to *Bacillus cereus* AlkD [33,38], the DNA glycosylase YtkR2 adopts a non-base-flipping mechanism to excise 3-yatakemycinyladenine nucleobase [33,37]. The resulting AP site (apurinic/apyrimidinic site) is proposed to be processed by proteins TtkR3, TtkR4, and TtkR5, which are homologous to the enzymes involved in the BER system, including xanthine phosphoribosyl-transferase, mental-dependent TatD family of DNase and AP endonuclease, respectively [39,40]. Together, these six proteins form a multiple self-defense network to protect the producer from YTM toxicity. Given that the homologs of YtkR2 and YtkR7 were found in the producing strain of CC-1065 [35,37], another compound in SCPCHD family, it is likely that this self-defense network is a generic self-resistance strategy for SCPCHD natural product biosynthesis.

AZB, isolated from the culture medium of *Streptomyces sahachiroi*, is a genotoxic antibiotic that displays excellent antibacterial and antitumor activity by forming interstrand crosslinks (ICLs) in the duplex DNA sequence 5′-d(GNPy)-3′ via alkylating the N7 nitrogen of purine residues [41]. The AZB-producer *S. sahachiroi* is known to be protected by at least four proteins encoded by resistance genes, *aziR*, *aziE*, *alkZ,* and *aziN*, via drug sequestration, efflux, target protection and DNA damage repair, respectively (Figure 3B) [42,43,44,45]. Among them, *alkZ* plays a dominant role because it is essential for AZB production [43]. AziR, which is a drug-binding protein switched from aminoglycoside phosphotransferase, is responsible for binding to AZB to shield the drug activity [42]. The transmembrane export protein encoded by *aziE* couples with the drug-binding protein AziR to form an effective efflux system to transport AZB outside of the cell, thereby maintaining a low concentration of the toxic compound [43,45]. Despite this, the intracellular residual drug might still cause DNA methylation to form AZB-adducts. In this case, glycosylase AlkZ of HTH_42 superfamily will confer the AZB resistance through target site protection and DNA damage repair. First, AlkZ nonspecifically binds to intact DNA with its helix-turn-helix motifs to block target sites. Upon the DNA ICLs are formed by AZB alkylation, glycosylase AlkZ binds to the damage sites structure-specifically and catalyzes the hydrolysis of N-glycosidic bond of AZB-adducts to remove the alkylated nucleobase, triggering the BER pathway to repair DNA damage [43]. This is the first proposal of DNA glycosylase involved in the ICLs damage repair in prokaryotes. Although it is evolutionarily unrelated to the glycosylases AlkD/YtkR2, which is responsible for excision of bulky lesions, the crystal structure revealed that the glycosylase AlkZ uses a similar DNA-binding architecture and non-base-flipping mechanism to excise AZB-adducts [44]. In addition to BER repair, later in 2020, He et al. discovered that the AZB-mediated ICLs can also be repaired by the structure-specific endonuclease AziN via triggering a nucleotide excision repair-like pathway [45]. To be clear, knocking out *aziN* does not completely abolish the production of AZB, indicating that the repair pathway mediated by endonuclease AziN plays a secondary role compared with the BER pathway triggered by AlkZ. However, it is unclear when and how to recruit the endonuclease AziN to the damaged sites. In addition, whether there are direct or indirect interactions between the four resistance mechanisms remains unclear.

TXNA and LLD, two representative antibiotics of trioxacarcin family, are genotoxic polyketide natural products with prominent antimalarial, antibacterial, and antitumor activity [46,47]. To withstand the genotoxicity of TXNA and LLD [48,49], their producing bacteria have evolved drug efflux system and DNA damage repair proteins (Figure 3C), which are encode by genes in the *txn* and *lld* biosynthetic clusters [50,51,52,53,54]. The hypothetical MSF transmembrane export proteins encoded by *txnRr1* and *txnRr2* are supposed to play roles in the efflux of TXNA, and the DNA glycosylases encoded by *txnU2* and *txnU4* act on the TXNA-guanine adducts to trigger the BER pathway, providing self-protection to TXN toxicity [54]. Similarly, the homologs of TxnRr1/Rr2 and TxnU2/U4 encoded by the biosynthesis gene cluster *lld* provide resistance to LLD for the producer [54]. Intriguingly, although both TxnU/LldU and AlkZ are monofunctional DNA glycosylases of HTH_42 family, the former has a distinct catalytic motif relative to AlkZ, resulting in that TxnU/LldU exhibits no activity toward other bulky and crosslinking DNA adducts beyond TXNA/LLD-DNA lesions. Based on the crystal structure of TXNA-DNA, TXNA can not only alkylate DNA to form the bulky adducts, but also intercalate into the duplex DNA and extrude the nucleobase near the alkylating site out of the helix, resulting in an increased helical twist [49]. It would be interesting to find whether TXNA/LLD-DNA lesions are also repaired by other enzymes or pathways, such as NER or endonuclease AziN reported in AZB biosynthesis [45]. Therefore, more work is needed to elucidate the self-resistance system of TXNs.

CMNs represent a mixture of nonribosomal peptide antibiotics including CMN IA, IB, IIA and IIB that are second-line drugs for the treatment of multidrug-resistant tuberculosis [55,56]. These molecules bind to the bacterial ribosomes and inhibit the biosynthesis of proteins [57]. CMN-producing strain *Saccharothrix mutabilis* subsp. *capreolus* possesses several self-resistance mechanisms to overcome the cytotoxicity (Figure 3D). The *cac* gene, located far away from the CMN BGC, encodes an N-acetyltransferase that inactivates CMN IIA and IIB via acetylation of the *β*-lysine moiety [58]. The second resistance gene was proposed to be *cmnU* within the BGC, deduced to encode a methyltransferase related to the KamB and KamC that confer resistance to kanamycin by methylating 16S rRNA. Consistent with this proposal, the expression of *cmnU* in *Streptomyces lividans* and *Escherichia coli* conferred high-level resistance to CMN and kanamycin [58], although an in vitro assay of CmnU for rRNA methyltransferase activity was not performed. The third is the *cph* gene that plays a role in the phosphorylation of CMN IA and IIA at the Ser hydroxyl group, but not on CMN IB or IIB. Subsequent biochemical assays and structure analysis determined that the Cph exhibits a high binding affinity to both CMN IIA and IIB, and its expression confers *E. coli* resistance to CMN IIB, suggesting Cph also serves as an antibiotic sequester beyond phosphotransferase [59]. Consequently, compared to general phosphotransferases, Cph adopts a dual mechanism to inactivate antibiotics by either chemical modification or physical sequestration. Besides drug modification and sequestration enzymes, the CMN BGC also contains a hypothetical MSF efflux protein that was deduced to transport the phosphorylated CMN, while the transportation has not been verified.

Colibactins are a group of genotoxic, nonribosomal peptide-polyketide secondary metabolites of gut-commensal *E. coli* that contain the *pks* island [60,61,62]. These cryptic genotoxins have attracted continuous studies due to their close correlation to human health. They cause DNA double-strand breaks through DNA crosslinking or copper-mediated oxidative cleavage, leading to cell-cycle arrest and even cell death [63,64,65]. To counter the cytotoxicity of colibactin, at least four colibactin-resistance determinants (*clbN*, *clbB*, *clbM* and *clbS*) are present in the *pks* biosynthetic cluster (Figure 3E). Nonribosomal peptide synthetases ClbN and ClbB install an N-acyl-D-asparagine prodrug motif at the N-terminus of colibactin to prevent the formation of the active imine moiety at the early stage of colibactin biosynthesis, thereby generating the inactive prodrug [66,67]. Subsequently, the resulting precolibactin will be transported into the periplasm by the 12-transmembrane MATE inner-membrane transporter ClbM [68,69,70], where mature colibactin is generated via cleavage of prodrug structure with peptidase ClbP, followed by out membrane translocation [67,71]. Notably, knocking out *clbM* does not completely abolish the production of colibactin, indicating that precolibactin can also be exported across the cytoplasmic membrane by other means. The intracellular cyclopropane hydrolase encoded by *clbS* abrogates cytotoxicity of the offloaded intermediates or colibactin by converting the electrophilic cyclopropane into an innocuous hydrolase product [72]. Interestingly, besides directly inactivating genotoxic cyclopropane, ClbS also functions as a DNA binding protein that protects the bacterial DNA from nucleolytic degradation [73,74]. However, the relationship between DNA binding activity and colibactin resistance remains obscure.

## 4. Resistance Widespread in Nature

**Figure 4:**
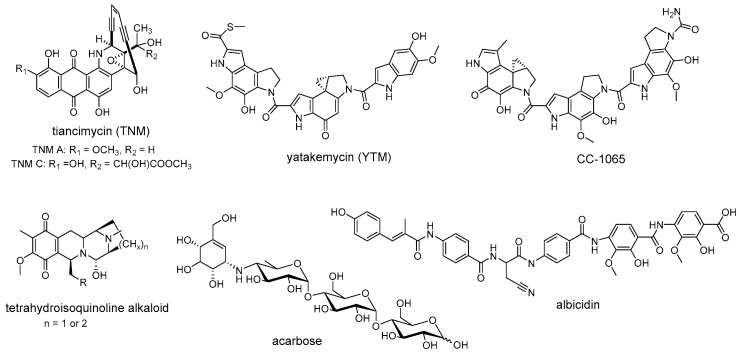
Representative natural products that resistance determinants are widespread in nature.

Self-resistance determinants are not confined to the antibiotic producers. Instead, some of them are widely prevalent in the clinical pathogens and environmental bacteria [75]. Recent studies of self-resistance mechanisms against enediyne antitumor antibiotics revealed that an unprecedented sequestration mechanism for the anthraquinone-fused enediynes has been evolved in their producers and the homologs of these resistance elements are widely distributed in nature [16]. Within the gene cluster of tiancimycin (Figure 4), resistance genes *tnmS1*, *tnmS2*, and *tnmS3* play a role in the sequestration of tiancimycin. The homologs of TnmS1, TnmS2, and TnmS3 are widespread in anthraquinone-fused enediynes producers and other bacteria, from different body sites, including the human microbiome [16]. The expression of homologous genes from the gene clusters encoding enediyne biosynthesis has been reported to endow *E. coli* BL21(DE3) with cross-resistance to anthraquinone-fused enediynes, while the homologs from human microbiome confer specific resistance to tiancimycin A [16]. These results further highlight that the resistance elements responsible for anthraquinone-fused enediynes sequestration are widely distributed in nature, although little is known about how these resistance genes disseminate in the environment. Similarly, homologous resistance genes encoding following enzymes are widespread in nature and perform conserved biological functions, such as GyrI-like cyclopropane hydrolases that mediate cyclopropyl moiety opening of DNA-alkylating agents YTM/CC-1065 [35], AlbA-like drug-binding proteins that guide resistance to albicidin [76,77], and NapW-like short-chain dehydrogenase/reductase that catalyze hemiaminal pharmacophore inactivation for tetrahydroisoquinoline antibiotics (Figure 4) [18]. In addition to antibiotic resistance, non-antibiotic drug resistance is also widespread. As direct evidence for this conception, Acbk-like kinase, inactivating a clinically used non-antibiotic antidiabetic drug acarbose by phosphorylation, is widely distributed in the human gut and oral microbiome (Figure 4) [78]. The specific kinase AcbK derived from *Actinoplanes* sp. SE50/110, is located within the gene cluster for acarbose synthesis. It phosphorylates acarbose at the O6A hydroxyl and serves as the self-resistance mechanism for acarbose production [79]. Recently, Donia et al. performed a metagenomics-based investigation of the human microbiome and found that homologues of AcbK are widespread in the bacteria from the human gut and oral microbiome and provide acarbose resistance, indicating the phosphorylation strategy of acarbose has disseminated in the human microbiome as a resistance mechanism [78]. Therefore, research on these widely distributed resistance elements will contribute to predicting and combating clinical drug resistance.

## 5. Resistance-Guided Natural Products Discovery

**Figure 5:**
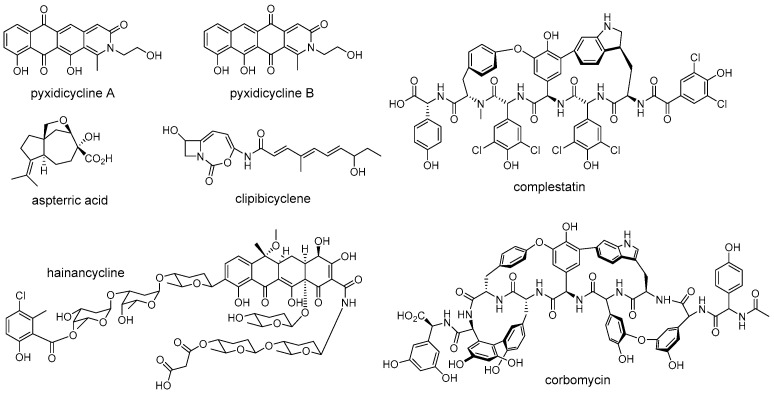
Structures of representative natural products that discovered by using antibiotic resistance gene as a probe.

The rapid development of bioinformatics tools and genome sequencing technologies has brought a revolution in the discovery of natural products, leading to a transformation from traditional bioactivity-guided fractionation to modern genome-based target mining [80,81]. The enormous amount of genome data that is now available has revealed that microorganisms harbor more natural product BGCs than those observed under laboratory cultivation conditions, and most of them gene clusters encoding unknown products [82,83]. However, how to deal with the increasing BGCs and how to mine the desired products from the huge resources have become a major focus. Recently, researchers found that the self-resistance genes co-localized with BGCs can be used as a potential tool to link BGCs with molecular targets for mining natural products with desired activity (Figure 5) [8,84,85]. For example, Tang and coworkers successfully discovered a natural herbicide with a new mode of action from *Aspergillus terreus* by a putative self-resistance gene, *astD*, encoding a dihydroxyacid dehydratase (DHAD) homolog [14]. The DHAD is an essential enzyme that catalyzes the last step of branched-chain amino acid biosynthesis, and is therefore effectively targeted for herbicide development [14]. However, no compounds that target this enzyme have been reported in planta. Fungal genomes scanning of a DHAD homologue revealed that a BGC encoding a sesquiterpene cyclase homologue and a DHAD homologue was present in the genome of *Aspergillus terreus*. Subsequent experiments demonstrated that the aspterric acid encoded by this BGC is indeed a competitive inhibitor of DHAD and effectively functions as a herbicide, and the DHAD variant AstD functions as a self-resistance enzyme in the BGC for aspterric acid [14]. Similarly, the Müller group discovered a novel group of topoisomerase inhibitors, including pyxidicycline A and B, by putative self-resistance genes encoding topoisomerase-targeting pentapeptide repeat protein [86]. Wright et al. identified the caseinolytic protease (ClpP) inhibitor clipibicyclene from *Streptomyces cattleya* using ClpP as putative antibiotic resistance gene [87]. Ge et al. discovered a novel tetracycline, hainancycline, by using the common tetracycline antibiotics resistance enzyme TetR/MarR-transporter as probe [88].

**Figure 6:**
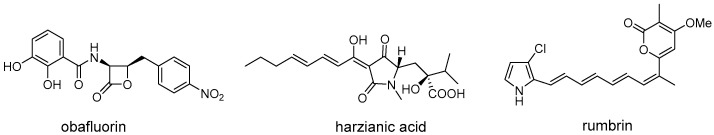
Structures of representative natural products that determine the biomolecular target through antibiotic resistance genes.

In addition to discovering natural products with desired activity, self-resistance genes can also be used in determining the biomolecular target of known antibiotics (Figure 6). For instance, the β-lactone obafluorin isolated from *Pseudomonas fluorescens* ATCC 39502 shows potent antibacterial activity against both Gram-positive and Gram-negative pathogens [89]. The mechanism of action of obafluorin, however, was unknown as this molecule was reported to cause an unusual cell-elongation phenotype compared to other β-lactone antibiotics. During comparative genomic analysis of obafluorin BGCs, an open reading frame, *obaO*, was identified and speculated to be an immunity gene [89]. ObaO was shown to be a homologue of threonyl-tRNA synthetase and conferred resistance to obafluorin-sensitive strains and obafluorin producer when expressed. Subsequently, in vitro enzyme assays demonstrated that the obafluorin did indeed fully inhibit *E. coli* threonyl-tRNA synthetase with an IC_50_ of 92 ± 21 nM, thus indicating the target of this compound [89]. In another example, harzianic acid is a N-methylated tetramic acid isolated from *Trichoderma harzianum* in 1994. Although it displays excellent antifungal activity, including against plant pathogens *Sclerotinia sclerotiorum* and *Rhizoctonia solani*, the molecular target of harzianic acid remains unknown [90]. Recently, Tang et al. discovered that the harzianic acid is an inhibitor of acetohydroxyacid synthase (AHAS, the first enzyme on branched-chain amino acid biosynthesis pathway), which was guided by a truncated AHAS homolog resided within the BGC that was demonstrated to be the self-resistance enzyme [91]. A similar biomolecular target discovery scenario is also observed in determining the mode of action of polyketide rumbrins, which further revealed their promising potential to be HIV inhibitors [92].

Although the above examples have successfully confirmed the potential of self-resistance genes in directed genome mining for natural products with known or predicted biomolecular targets, the development of compounds with novel mechanisms of action is also an urgent need to solve the ongoing antibiotic crisis. Recently, Wright et al. reported that the method of combining the absence of known self-resistance genes with phylogenetic analysis of biosynthetic genes could be effective in finding natural products with new modes of action [93]. They applied this approach to the glycopeptide family of antibiotics and successfully discovered a novel functional class of glycopeptide antibiotics composed of complestatin and corbomycin (Figure 5), which have a new mechanism of action that inhibits peptidoglycan re-modelling. This research outcome again indicated that self-resistance determinants are useful for prioritizing BGCs than just function in the self-protection. Other examples of employing a self-resistance determinant in natural products discovery are reviewed elsewhere [6,8,94]. Taken together, self-resistance genes can be a bridge between the bioactivity-guided and genome-based methods for natural products discovery. Studying the complex self-resistance strategies from a temporal-spatial shielding perspective will allow researchers to further understanding the evolutionary relationship between natural product biosynthesis and resistance, thereby facilitating discovery of new drug candidates with high activity.

## 6. Conclusions and Perspective

Organisms that produce toxic natural products have evolved various self-resistance determinants in offensive and defensive contexts. According to the location where final toxic compounds are produced, we summarized two models that organisms utilize for self-protection, intending to provide a perspective on the ties between toxic antibiotic biosynthesis and self-resistance from spatial distribution. Research on self-resistance mechanisms extend well beyond revealing the origin of natural product resistance and predicting the action model of these molecules. In terms of bioengineering, a deep understanding of antibiotic resistance mechanisms is crucial for the efficient synthesis of target natural products and the development of next-generation antibiotics capable to overcome established clinical resistances. Additionally, determinants conferring self-protection in antibiotic producing organisms are considered to represent a major reservoir of resistance genes, which could disseminate into human pathogens by horizontal gene transfer [13]. Reviewing self-resistance with a multi-dimensional perspective is therefore helpful to better cope with the increasing drug resistance in clinical settings. Moreover, regarding natural products mining, self-resistance proteins mutated from essential housekeeping enzymes have effectively served as a tool to link BGCs with molecular targets. However, using this strategy for genome mining might inevitably lead to the mining of results skewed to known compounds. The continued study of natural product biosynthesis and multi-dimensional self-resistance is required for mining desired compounds with higher accuracy. Taken together, self-resistance genes are expected to serve as models to predict and combat drug resistance in clinical settings and to be an effective bridge between the bioactivity-guided and genome-based methods for natural product discovery, thus facilitating discovery of new drug candidates. It is expected that more self-resistance determinants will be discovered to enrich our knowledge on the relationship between natural product toxicity and resistance genes and facilitate the discovery of new drug candidates to combat clinical resistance.

## Data Availability

Not applicable.
